# Surface-Related Exciton and Lasing in CdS Nanostructures

**DOI:** 10.1186/s11671-019-3036-5

**Published:** 2019-06-25

**Authors:** Xian Gao, Guotao Pang, Zhenhua Ni, Rui Chen

**Affiliations:** 1 Electrical and Electronic Engineering, Southern University of Science and Technology, Shenzhen, Guangdong 518055 People’s Republic of China; 2 School of Physics, Southeast University, Nanjing, 211189 People’s Republic of China

## Abstract

In this report, comparative investigation of photoluminescence (PL) characteristics of CdS nanobelts (NBs) and nanowires (NWs) is presented. At low temperatures, emissions originate from radiative recombination of free exciton A, neutral donor bound exciton, neutral acceptor bound exciton and surface related exciton (SX) are observed and analyzed through power-dependent and temperature-dependent PL measurements. We found that SX emission takes a predominant role in emissions of CdS nanobelts and nanowires. There is a direct correlation between SX emission intensity and surface-to-volume ratio, which is the SX emission intensity is proportional to the superficial area of the nanostructures. At the same time, we found that the exciton-phonon interaction in the CdS NWs sample is weaker than that of CdS NBs sample. Furthermore, lasing action has been observed in CdS NBs sample at room temperature with lasing threshold of 608.13 mW/cm^2^. However, there is no lasing emission in CdS NWs sample. This phenomenon can be explained by the side effects (such as thermal effects) from surface deep level transitions caused the lower damage threshold in CdS NWs. Based on the observations and deductions presented here, SX emission significantly impact on the performance of nanostructures for lasing and light-emitting applications.

**Electronic supplementary material:**

The online version of this article (10.1186/s11671-019-3036-5) contains supplementary material, which is available to authorized users.

## Background

Low-dimensional nanomaterials play an important role in photonic devices. Many research have been carried out to characterize their unprecedented properties derived from their quantum size in at least one dimension or strong anisotropy [1–4]. The richness of nanostructures facilitates the observation of various interesting phenomena, which allows the integration of functional nanomaterials into a wide range of applications. Due to the large surface-to-volume ratio, the optical properties of low-dimensional semiconductors are strongly affected by the material quality and surface morphology. Up to date, various low-dimensional semiconductors are used in micro/nano-devices, such as CdS, ZnO, ZnS, and GaAs, etc. [5–7]. As one of the most important applications, laser devices with low threshold, high reliability, and good stability are highly desired. In the past decade, research on the nanostructure-based laser devices has focused on the ability to generate lasers due to their optical gain media and natural optical cavities [1].

CdS is an important II–VI group semiconductor with a direct band gap of 2.47 eV at room temperature, which can be used as high-efficient optoelectronic material in the ultraviolet-visible range. So far, a large number of CdS nanostructures have been synthesized successfully, such as nanospheroids, nanorods, nanowires, nanotripods, nanocombs, and nanobelts [8]. In addition, low-dimensional CdS nanostructures have been proven to have potential applications in nano-optoelectronic devices, such as visible range photodetection [9], optical refrigeration [10], waveguide, and laser devices [11, 12]. In recent years, lasing phenomena in CdS nanobelts (NBs) and nanowires (NWs) have been discovered and studied [13–17]. It is worth noting that large surface-to-volume ratio and quantum confinement effects can strongly influence the band gap, density of states, and carrier dynamics in the low-dimensional CdS nanostructures. In this case, the influence of the surface state on carriers and phonons is also increasing. It can be proven that lattice vibration and excitons can be localized on the surfaces of nanostructures and can be called surface optical phonon mode [18, 19] and surface-related exciton, respectively. Surface excitons could be one kind of excitons bound at surface state, which could be related to Tamm states [20] and surface defects [21–23].

Therefore, the carrier dynamics of low-dimensional CdS nanostructures become more complex than bulk and thin film materials due to the surface states, thermal effect, and surface depletion [24, 25]. Although the optical properties of CdS nanostructures have been extensively studied by other researchers, the current understanding of the surface exciton and related lasing mechanisms is still far more complete. It is necessary to conduct detailed carrier kinetic studies on surface exciton to understand the mechanism of photoelectron properties in nanoscale materials for further application [26].

In this work, a systematic comparison of the optical properties of CdS NBs and NWs was performed. Surface states-related exciton emission in nanostructures is discussed by analyzing their photoluminescence (PL). High-density optical pumping experiments are used to clarify the effect of surface-to-volume ratio on lasing. Our results indicate that the surface states-related exciton in CdS nanostructures take an important role in its optical properties, and the associated lasing emission can be obtained at room temperature. These results also reveal the influence of quantum confinement effect and exciton-LO-phonon interaction in CdS NBs and NWs.

## Methods

### Material Growth

The CdS NBs and NWs were synthesized from pure CdS nanopowder (Alfa Aesar CdS powder) by physical evaporation using a solid tube furnace (MTI-OFT1200). The CdS NBs and CdS NWs were grown on Si (100) wafers, which were cut into 1 cm^2^ before the experiment. According to the SEM results, the CdS NB has a width of about 1 μm and a thickness of about 70 nm, and the diameter of the CdS NWs is about 90 nm (as shown in Additional file 1: Figure S1).

### Optical Characterization

All PL spectral signals were dispersed by an Andor spectrometer, combined with a suitable optical filter, and then detected by a charge-coupled device (CCD) detector. A He-Cd laser with laser line of 325 nm was used as the excitation source for temperature and power-dependent PL measurements. For the optical pumping experiment, a pulsed 355 nm laser with a pulse width of 1 ns and a frequency of 20 Hz was employed as the excitation source. For the temperature-dependent PL measurement, the sample was mounted inside a helium closed-cycle cryostat (Cryo Industries of America), and the temperature of sample is controlled by a commercial temperature controller (Lakeshore 336 temperature controller). In the excitation power-dependent PL measurement, a variable neutral density filter was used to obtained different excitation power densities. To ensure comparability of PL results, optical alignment is fixed during the measurement.

## Results and Discussion

**Fig. 1:**
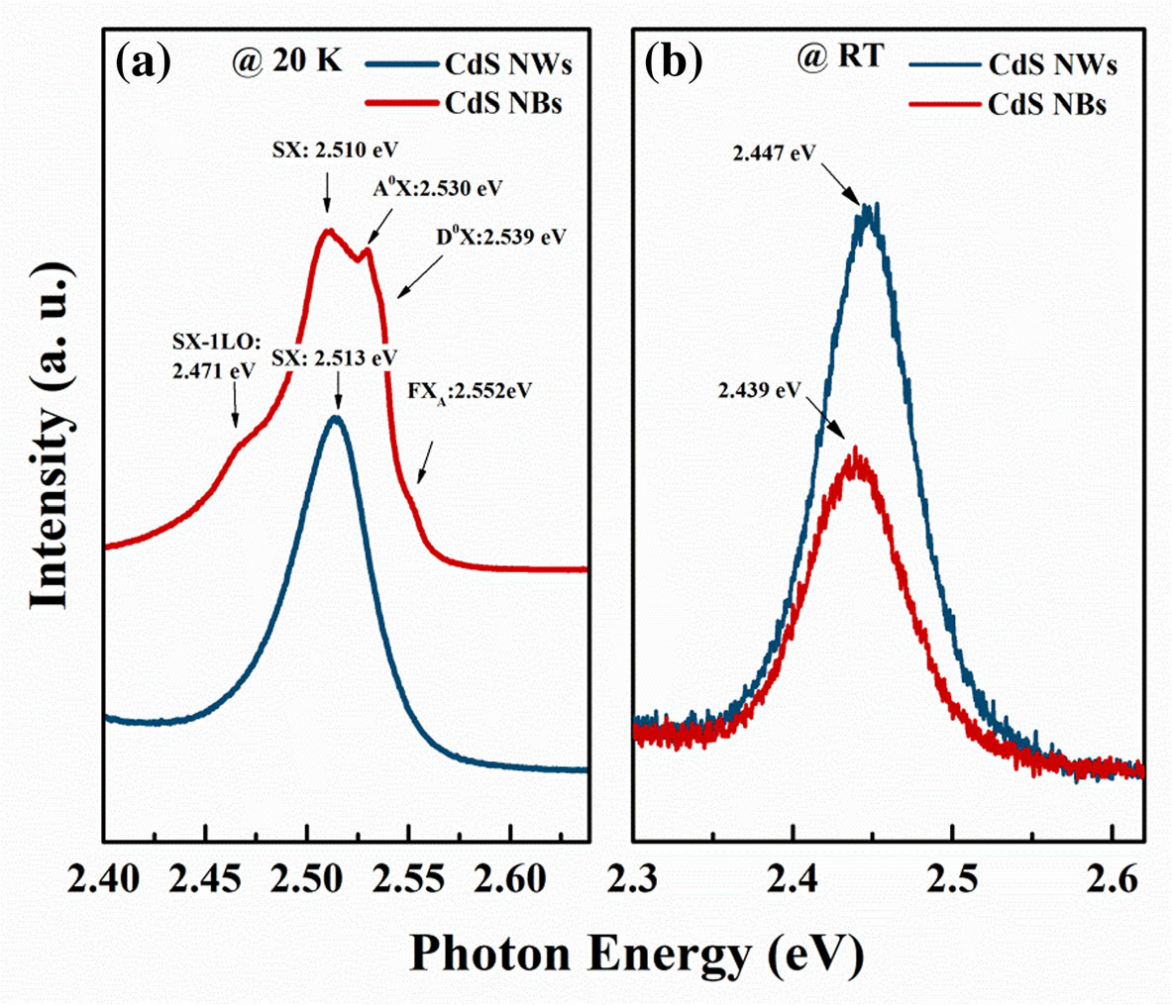
The PL spectra of CdS NBs and NWs (**a**) at 20 K and (**b**) at room temperature

Figure 1 shows the low temperature (20 K) and room temperature PL spectra of CdS NBs and NWs samples. These PL spectra were all measured at an excitation power of 8 mW. For clarity, the PL spectral data in Fig. 1a is normalized and vertically offset. It can be seen that the spectrum of CdS NBs displays some exciton emission-related structures. The corresponding peaks located at 2.552, 2.539, and 2.530 eV can be labeled as free exciton A (FX_A_), neutral donor-bound exciton emission (D^0^X) and neutral acceptor bound exciton (A^0^X), respectively. These peaks can be reasonably assigned according to their characteristic emission energy [12, 27]. Significantly, we assume the emission at 2.510 eV is surface states-related exciton emission and labeled it as SX, and the detailed results will be discussed later. As is known, the surface-related exciton is a kind of bound exciton, which is associated with surface-related defects, such as the study of surface exciton in ZnO and other nanostructures [18–20]. Considering the energy of longitudinal optical (LO) phonon of CdS is about 38 meV, the lower energy side peak (2.471 eV) could be assigned to the first order LO phonon replica of SX. In contrast, CdS NWs sample showed an asymmetric emission peak with a peak position at 2.513 eV. This peak also can be assigned to the recombination of the surface states-related exciton (SX). Figure 1b displays the room temperature PL spectra of CdS NBs and NWs. Compared with CdS NBs, the peak position of SX shows a little blue shift. It is worth mentioning that the SX emission intensity of CdS NWs sample is about two times higher than that of CdS NBs sample. The CdS NWs sample has a larger surface-to-volume ratio than the CdS NBs sample, so the luminescence of the two nanostructures at room temperature could be related to surface, that is, related to surface exciton. Considering the SEM result in Additional file 1: Figure S1, we found it is difficult to find bared Si substrate in CdS NBs image, instead, a bared substrate can be seen in CdS NWs sample. This result means that the coverage of the CdS NBs sample per unit area is much larger than that of the CdS NWs sample (as shown in Additional file 1: Figure S1). At the same time, under the same measurement conditions, the reflection intensity of the laser in CdS NWs is 8.2 times that of CdS NBs. Therefore, CdS NWs samples should have higher PL efficiency, which is consistent with the speculation that PL emission is related to surface excitons.

**Fig. 2:**
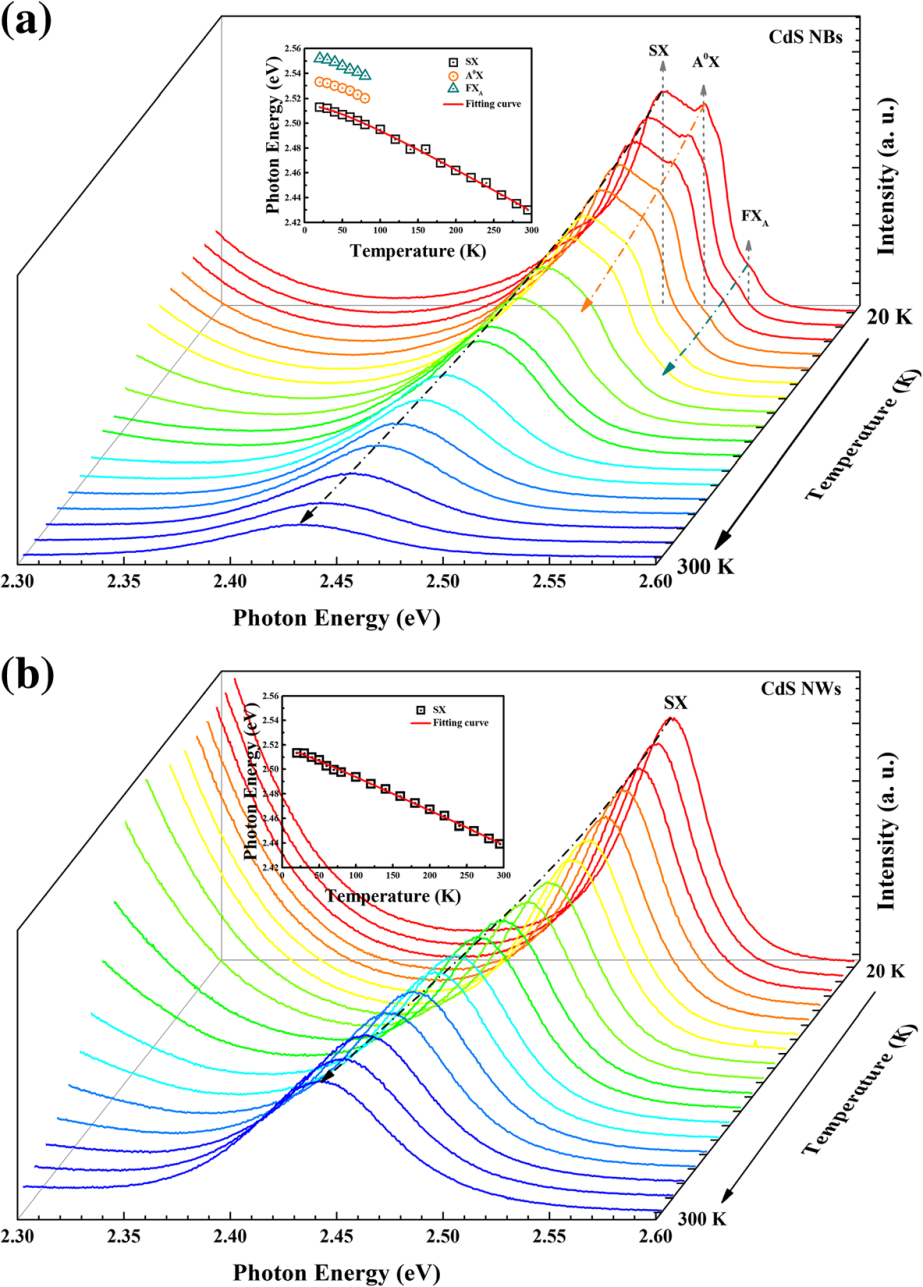
**a** Temperature-dependent PL spectra of CdS NBs in the range from 20 K to 295 K, the inset is plots of FX_A_, A^0^X, and SX peaks as a function of temperature. **b** Temperature-dependent PL spectra of CdS NWs in the range from 20 K to 295 K, the inset is SX peak redshift with the temperature, and the solid red curve of the SX is corresponding to the fit result based on Varshni equation

To reveal the evolution of the emission in CdS NBs and NWs samples, the temperature-dependent PL spectra were outperformed and analyzed. As depicted in Fig. 2a, the peaks of FX_A_, D^0^X, and A^0^X, all exhibit redshift with the increase of temperature, while in CdS NBs sample, SX emission dominates the emission on the temperature range of 20 to 295 K. The results show that the emission intensity of FX_A_, D^0^X, and A^0^X emission drops dramatically when the temperature rises, and their relative intensity decreases much faster than SX and disappears at around 100 K. The inset of Fig. 2a shows the plots of these peak positions evolution with the temperature. To understand the emission mechanism behind the PL results, we use the following empirical formula to describe the temperature induced bandgap shrinking [28]:

1 $$ {E}_g(T)={E}_g(0)-\frac{\alpha \Theta}{\exp \left(\raisebox{1ex}{$\Theta $}\!\left/ \!\raisebox{-1ex}{$T$}\right.\right)-1} $$

where *E*_*g*_(0) is the bandgap at 0 K, *α* is coupling constant between the electron (or exciton) and phonon which is associated with the strength of exciton-phonon interaction, Θ is an averaged phonon energy, and *T* represents absolute temperature. The symbols in the inset of Fig. 2a are experimental data of FX_A_, D^0^X, and SX, and the solid lines represent the fitting curves of SX. In this case, SX shows redshift with the temperature increase, and it can be well fitted by the above formula. This result indicates that SX is near band gap radiative recombination. The fitting parameter *E*_*g*_(0) of SX is approximately 2.512 eV in CdS NBs sample, which is located on the low energy side of FX_A_ peak. The energy difference between SX and FX_A_ is about 42 meV. The SX emission is gradually dominant when temperature rises, which also supports the SX emission attributable to a strong exciton.

In comparison, the temperature-dependent PL spectra of CdS NWs are shown in Fig. 2b. It can be seen that the PL spectrum shows only one emission peak in the temperature range of 20 to 295 K. This peak located at 2.513 eV at 20 K, and it should be assigned to SX emission. This SX peak position is also well fitted by Eq. 1, which also confirmed SX emission is related to the near band gap transition. The parameter of the fitting results for CdS NBs and NWs are collected in Table 1. The difference value of *Eg*(0) between CdS NBs and NWs is 3 meV. Evidently, the exciton-phonon coupling constant *α* and averaged phonon energy *Θ* of the CdS NWs are smaller than those of the CdS NBs. This result also suggests that weakened exciton-LO-phonon coupling exists in the CdS NWs sample, which is caused by the long-range translational symmetry was partly destroyed [28].

**Table 1:** The fitting parameters for CdS NBs and NWs samples

Parameter Sample	*E*_*g*_(0) (eV)	*α* (eV K^−1^)	Θ (K)
SX (CdS NBs)	2.512	3.314×10^−4^	105.6
SX (CdS NWs)	2.515	2.757×10^−4^	46.2

Next, for better understanding the optical characteristics of SX emission in CdS NBs and NWs samples, power-dependent PL measurement was carried out

**Fig. 3:**
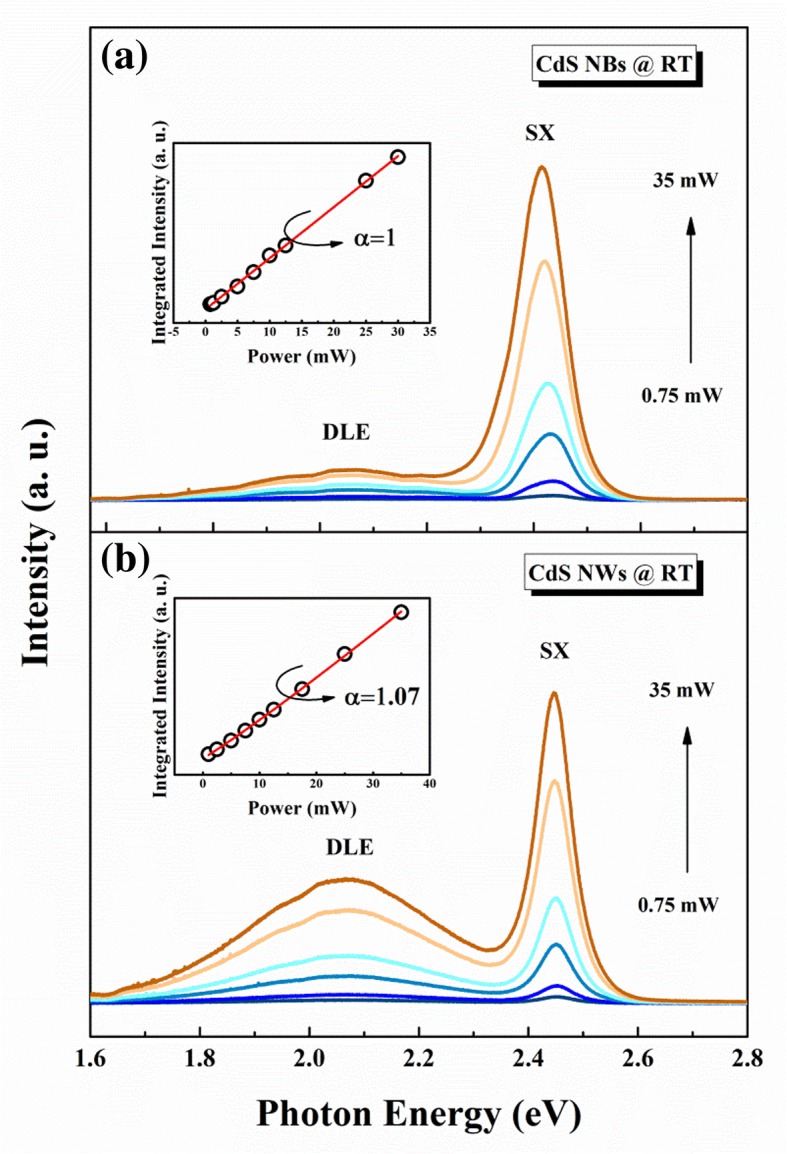
**a** PL spectra of CdS NBs under different excitation power at room temperature, the inset is the integrated intensities of SX with the excitation power. **b** PL spectra of CdS NWs under different excitation power at room temperature, the inset is the integrated intensities of SX with the excitation power

Figure 3a presents the power-dependent PL spectra of CdS NBs sample at room temperature. The emission peak at 2.44 eV is the radiative recombination of SX, while an emission band centered at 2.06 eV may be derived from the deep level defects such as Cd interstitial, dangling bonds, surface defects, or S vacancies [29–31]. The relationship between excitation power *I*_*0*_ and integrated intensity of emission *I* can be expressed as following [32]:

2 $$ I=\eta {I}_0^{\alpha } $$

where *I*_*0*_ is the power density of the excitation, *η* represents the emission efficiency and the exponent *α* indicates the mechanism of the recombination. The intensity of the emission peak keeps growing with the excitation power increasing. The inset of Fig. 3a depicts the PL intensity of SX emission in CdS NBs as a function of laser power density, and the solid line present the fitting result of Eq. 2. For SX emission, the exponent α is about 1, which indicate SX emission is still excitonic recombination at room temperature.

In contrast to CdS NBs results, deep level emission (DLE) is more obvious in CdS NWs sample (as shown in Fig. 3b). This can be explained as CdS NWs have more surface defects due to its larger surface-to-volume ratio. The inset of Fig. 3b gives the integrated PL intensity plots as a function of excitation power, which can be fitted by Eq. 2. The fitting parameter *α* of CdS NWs sample is equal to 1.07, which also supports SX emission to be of exciton nature.

**Fig. 4.**
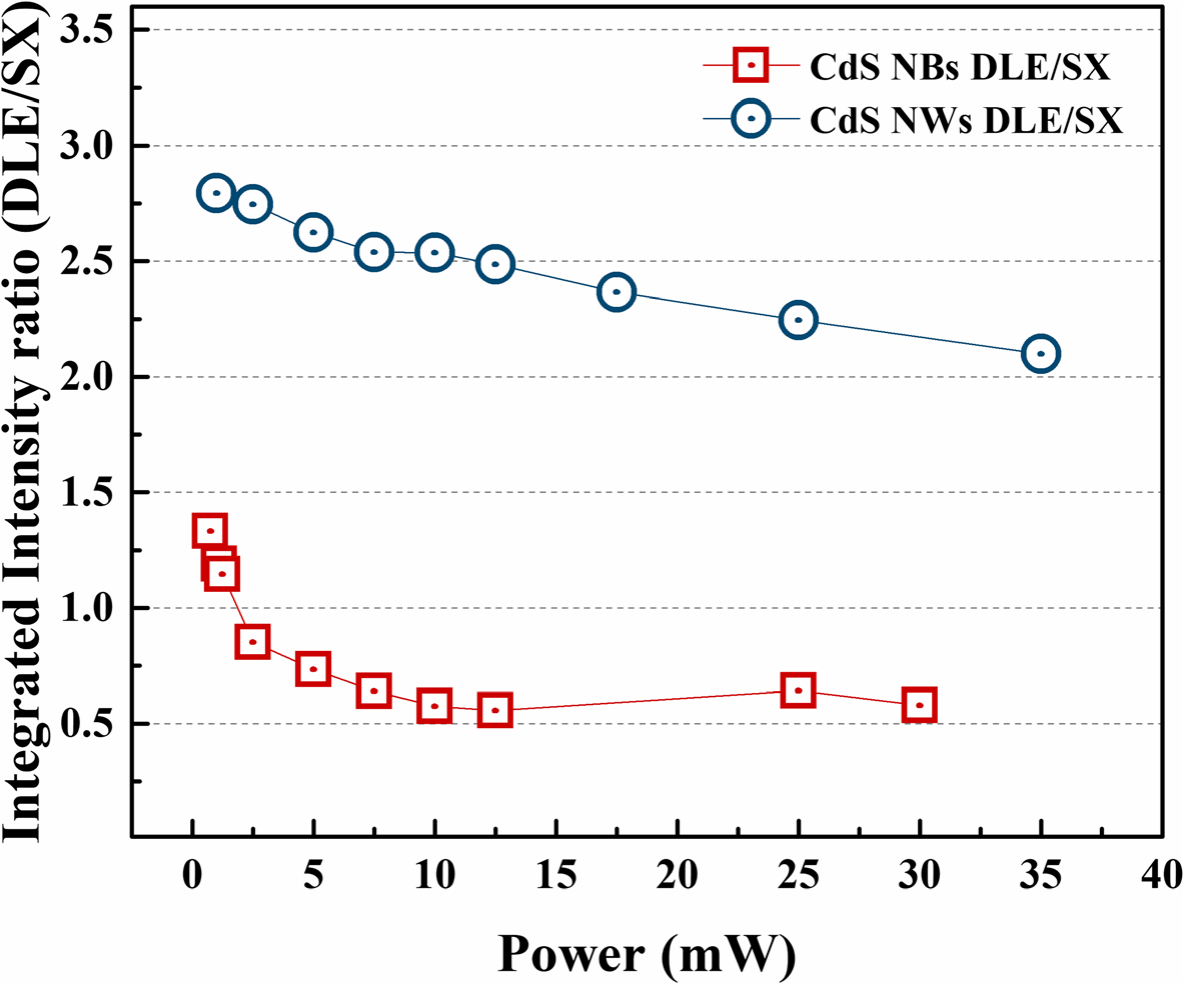
Integrated PL intensity ratio of DLE emission and SX emission in CdS NBs and NWs samples at room temperature

Figure 4 displays the integrated PL intensity ratio of DLE and SX emission in CdS NBs and NWs sample, respectively. It is clearly that DLE in CdS NBs take a dominant role in PL spectra at low excitation condition because of the DLE/SX is higher than 1. Then, the value is decreased with the enhancement of excitation power, which means SX emission have higher rise ratio than DLE emission. On the other hand, the DLE of CdS NWs sample show a higher ratio up to 2.8 and dropped slowly with the raised excitation power. This result confirmed DLE emission dominated the spectra in CdS NWs. Although the larger surface-to-volume ratio can induce more SX emission, but DLE also became higher at the same time. It is clear that more carriers in higher energy states will first relax to DLE states and then do radiative recombination (DLE emission) in CdS NWs sample. The general side effect of the DLE emission is thermal effects, thus, it may influence the optical properties of CdS NBs and NWs.

**Fig. 5.**
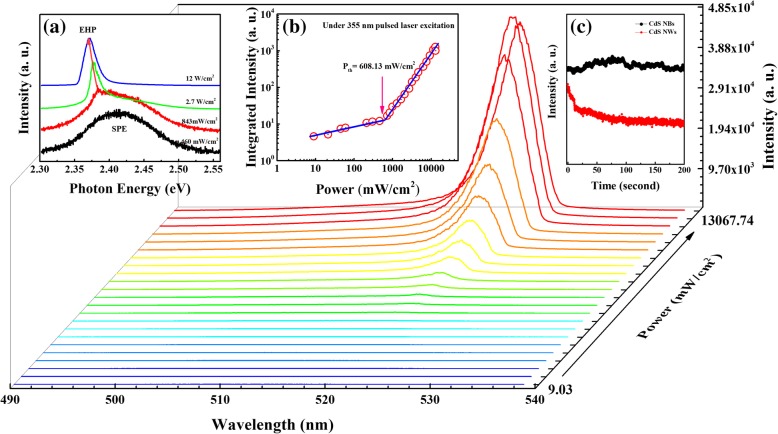
Power-dependent lasing spectra of CdS NBs at room temperature, the inset **a** shows the trend of lasing emission peak, the inset **b** is integrated peak intensity as a function of excitation power, and the inset **c** represents the PL intensity of CdS NBs and NWs plots as a function of time, both samples excited under 355 nm pulsed laser with the power density of 12.8 W/cm^2^

Next, a 355-nm pulsed laser is used as excitation source to investigate the lasing action in CdS nanostructures. Figure 5 shows the power-dependent PL spectra of CdS NBs at room temperature. To obtain the lasing threshold, integrated PL intensities are plotted as a function of the average power density as shown in Fig. 5b. A superlinear increase of emission intensity and sharp features occurred when the average power density is about 608.13 mW/cm^2^. And the instantaneous power intensity of the lasing threshold is 3.04 GW/cm^2^. With further increases of pump density, the center of lasing peak has a trend of redshift (as shown in Fig. 5a), which suggest the lasing peak could be ascribed to electron-hole plasma (EHP) recombination [33, 34]. However, when the power density increases above 13 W/cm^2^ or more, the intensity of lasing peak tends to decrease. If further increase the power density, sample will be damaged at the excitation laser spot. It can be ascribed to the thermal effect raised with the pump density.

Unfortunately, there is no lasing action that can be observed in CdS NWs sample. It is worth to mention that the damage threshold of the CdS NWs sample is about 2.65 mW/cm^2^, which is much lower than the lasing threshold in CdS NBs sample. This result can be ascribed by the side effect (thermal effects) of the massive DLE emission in CdS NWs. In the interest of observing the lasing emission stability in CdS NBs and SX emission stability in CdS NWs, Fig. 5c depicts the PL intensity of the two samples as a function of time (from 0 to 200 s) under the excitation power of 12.8 W/cm^2^. The CdS NBs sample showed stable laser emission, while the CdS NWs showed PL emission, and the PL intensity rapidly decreased with time from the beginning.

These PL results mean the SX-related lasing emission is stable in CdS NBs sample, but a lower damage threshold to limit the emission performance in CdS NWs sample. In our case, the SX-related lasing emission could be enhanced by the larger surface-to-volume ratio, but the side effects (such as thermal effects) from surface deep level transitions could become a critical issue to hinder their lasing application.

## Conclusions

In conclusion, we have investigated the PL properties of CdS NBs and NWs by using temperature and power-dependent PL spectra. CdS NBs sample displays more detailed spectral structure than CdS NWs sample at 20 K. With the temperature increasing, the intensities of other emissions (such as FX_A_, A^0^X, and D^0^X) faded around 100 K, while SX emission (surface state-related exciton emission) is mainly governed by the PL broadening SX emission as can be observed. And we found that the exciton-LO-phonon interaction effect in CdS NWs sample is weak than that of CdS NBs, which caused the breakage of long-range translational symmetry.

It is worth to note that the stable lasing emission can be observed in CdS NBs sample at room temperature, and the lasing threshold is about 608.13 mW/cm^2^ (average power density). However, there are no signs of lasing emission in CdS NWs sample. This may be due to its relative larger surface-to-volume ratio that increases side effects, such as thermal effects from surface deep level transition. These results also proved SX emission in CdS nanostructures can provide a convenient and high-efficiency channel for potential laser and light-emitting applications.

## Additional file

Additional file 1: **Figure S1**. SEM image of a CdS NBs sample and b CdS NWs sample are presented. (DOCX 477 kb)

## Data Availability

The authors declare that materials and data are promptly available to readers without undue qualifications in material transfer agreements. All data generated in this study are included in this article.

## Abbreviations

A^0^X: Neutral acceptor bound exciton
CCD: Charge-coupled device
D^0^X: Neutral donor bound exciton
DLE: Deep level emission
FX_A_: Free exciton A
LO phonon: Longitudinal optical phonon
NBs: Nanobelts
NWs: Nanowires
PL: Photoluminescence
SX: Surface-related exciton
