# Supplementary material for: Surface-Related Exciton and Lasing in CdS Nanostructures
Source: Nanoscale Res Lett. 2019 Jun 25;14:216. doi: 10.1186/s11671-019-3036-5 (PMC6592998; doi:10.1186/s11671-019-3036-5)

*Gao* *et al. Nanoscale Research Letters* (2019)
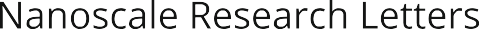


NANO EXPRESS

Open Access

Surface Related Exciton and Lasing in CdS
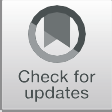
Nanostructures

Xian Gao^1,2^, Guotao Pang^1^, Zhenhua Ni^2, *^ and Rui Chen^1, *^

# **Supplemental Information**


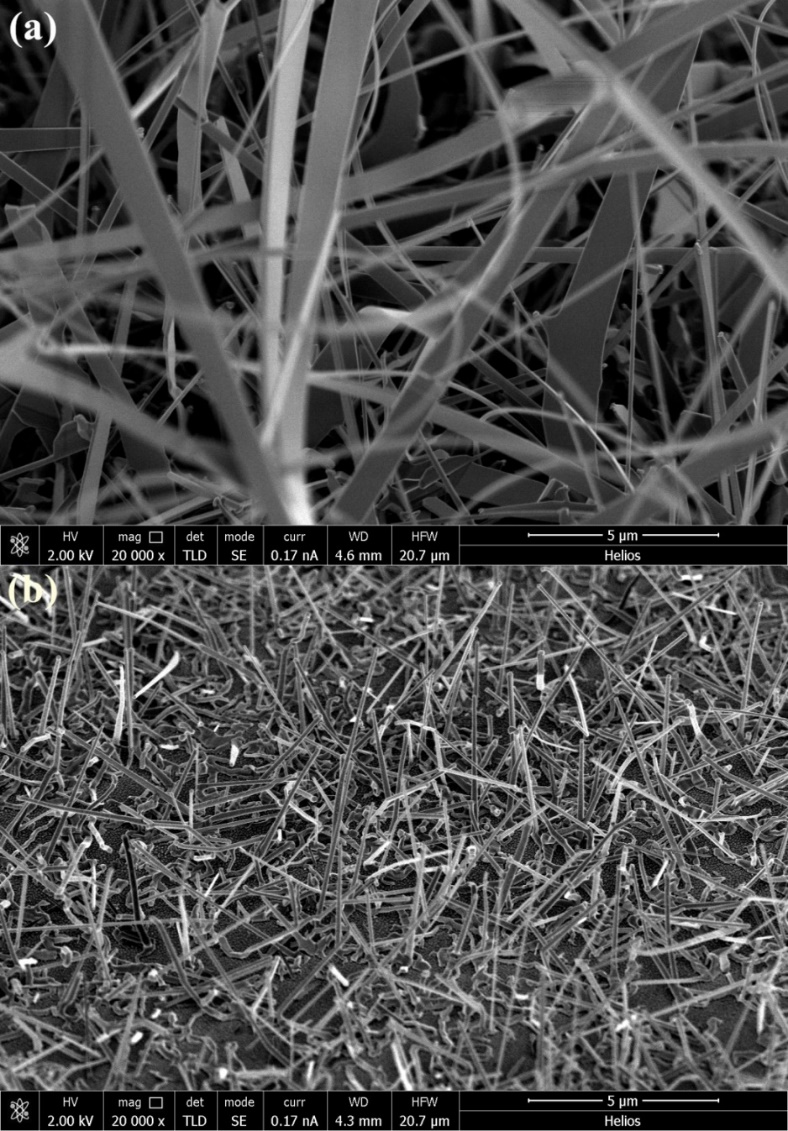


**Fig. S1** SEM image of (a) CdS NBs sample and (b) CdS NWs sample are presented.

The morphological evolution of CdS NBs and NWs were measured by using emission scanning electron microscopy (SEM). The width of the CdS NBs is about 1 μm and the thickness is about 70 nm. The diameter of CdS NWs is about 90 nm. In this Figure, the cover area of CdS NBs sample can be observed much larger than CdS NWs sample in the same area.

* Correspondence: [zhni@seu.edu.cn](mailto:zhni@seu.edu.cn); [chenr@sustech.edu.cn](mailto:chenr@sustech.edu.cn)

Co-author Email: [gaox@sustech.edu.cn](mailto:gaox@sustech.edu.cn); [panggt2018@mail.sustech.edu.cn](mailto:panggt2018@mail.sustech.edu.cn)

^1^ Electrical and Electronic Engineering, Southern University of Science and Technology, Shenzhen, Guangdong 518055, P. R. China

^2^ School of Physics, Southeast University, Nanjing 211189, P. R. China


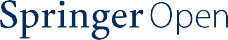


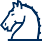

Supplement: Supplementary file 1 — Figure S1. SEM image of a CdS NBs sample and b CdS NWs sample are presented. (DOCX 477 kb) [file 11671_2019_3036_MOESM1_ESM.docx]
